# Development of Genome-Wide SSR Markers from *Angelica gigas* Nakai Using Next Generation Sequencing

**DOI:** 10.3390/genes8100238

**Published:** 2017-09-21

**Authors:** Jinsu Gil, Yurry Um, Serim Kim, Ok Tae Kim, Sung Cheol Koo, Chinreddy Subramanyam Reddy, Seong-Cheol Kim, Chang Pyo Hong, Sin-Gi Park, Ho Bang Kim, Dong Hoon Lee, Byung-Hoon Jeong, Jong-Wook Chung, Yi Lee

**Affiliations:** 1 Department of Industrial Plant Science & Technology, Chungbuk National University, Chungju 28644, Korea; kjs8789@chungbuk.ac.kr (J.G.); sese7510@nate.com (S.K.); jwchung73@chungbuk.ac.kr (J.-W.C.); 2 Forest Medicinal Resources Research Center, National Institute of Forest Science, Yeongju 36040, Korea; urspower@korea.kr; 3 Department of Herbal Crop Research, National Institute of Horticultural and Herbal Science, Rural Development Administration, Eumseong 27709, Korea; kimot@korea.kr (O.T.K.); ksch992@korea.kr (S.C.K.); suumani@gmail.com (C.S.R.); 4 Research Institute of Climate Change and Agriculture, National Institute of Horticultural and Herbal Science, Rural Development Administration, Jeju 63240, Korea; kimsec@korea.kr; 5 TheragenEtex Bio Institute, Suwon 16229, Korea; changpyo.hong@theragenetex.com (C.P.H.); singi.park@theragenetex.com (S.-G.P.); 6 Life Sciences Research Institute, Biomedic Co., Ltd., Bucheon 14548, Korea; hobang@ibiomedic.co.kr; 7 Department of Biosystems Engineering, Chungbuk National University, Chungju 28644, Korea; leedh@chungbuk.ac.kr; 8 Korea Zoonosis Research Institute, Chonbuk National University, Iksan 54531, Korea; bhjeong@jbnu.ac.kr

**Keywords:** *Angelica gigas* Nakai, next generation sequencing (NGS), simple sequence repeat (SSR)

## Abstract

*Angelica gigas* Nakai is an important medicinal herb, widely utilized in Asian countries especially in Korea, Japan, and China. Although it is a vital medicinal herb, the lack of sequencing data and efficient molecular markers has limited the application of a genetic approach for horticultural improvements. Simple sequence repeats (SSRs) are universally accepted molecular markers for population structure study. In this study, we found over 130,000 SSRs, ranging from di- to deca-nucleotide motifs, using the genome sequence of Manchu variety (MV) of *A. gigas,* derived from next generation sequencing (NGS). From the putative SSR regions identified, a total of 16,496 primer sets were successfully designed. Among them, we selected 848 SSR markers that showed polymorphism from in silico analysis and contained tri- to hexa-nucleotide motifs. We tested 36 SSR primer sets for polymorphism in 16 *A. gigas* accessions. The average polymorphism information content (PIC) was 0.69; the average observed heterozygosity (*H_O_*) values, and the expected heterozygosity (*H_E_*) values were 0.53 and 0.73, respectively. These newly developed SSR markers would be useful tools for molecular genetics, genotype identification, genetic mapping, molecular breeding, and studying species relationships of the *Angelica* genus.

## 1. Introduction

The genus *Angelica*, which includes more than 60 species, is an important medicinal herb in Far East countries, especially in Korea, Japan, and China. Many of these species have been used in traditional medicine to treat many diseases in Asian and Western countries [1]. *Angelica* species that grow naturally in South Korea, beside *Angelica gigas* Nakai, include *Angelica acutiloba* Kitagawa, *Angelica archangelica* L., *Angelica atropurpurea* L., *Angelica dahurica* (Fisch. ex Hoffm.) Benth. & Hook. f. ex Franch. & Sav., *Angelica decursiva* (Miq.) Franch. & Sav., *Angelica genuflexa* Nutt. ex Torr. & A. Gray, *Angelica glauca* Edgew., *Angelica jaluana* Nakai, *Angelica japonica* A. Gray, *Angelica koreana* L., *Angelica sinensis* Diels, *Angelica sylvestris* L., and others.

The root of *A. gigas* contains active compounds, such as decursin, and decursinol angelate [2], which is successfully used in treating gynecological and anemic health issues [3,4]. They are also used for inducing immune system, and for constipation relief [1,5]. Herbalists consider it equivalent to ginseng due to its remedial properties for several diseases including treating cancer [6], an anti-oxidant [7], neuro-protective, anti-flu, anti-hepatitis [1], and anti-bacterial activities [8]. The *A. gigas* root also contains important chemical compounds like pyranocoumarins, essential oils, and poly-acetylenes.

Due to the scarcity of efficient molecular markers, there is a dearth of information about the genetic relationships and genetic diversity among breeding populations of *Angelica* crops [9]. Thus, to address these issues, during the past 30 years many kinds of markers have been developed and used for crop genetics and breeding [10]. The improvement in DNA sequencing and genotyping in conjunction with the development of computer programs have helped the design of molecular markers such as simple sequence repeats (SSRs) in minor crops [11].

Molecular markers are widely used in various areas such as genetic-diversity characterization, gene-flow studies, quantitative-trait locus (QTL) mapping, and evolutionary studies [12]. Accumulated plant genome sequences have accelerated single nucleotide polymorphism (SNP) marker development. Numerous SNP markers have been successfully used to develop various crop cultivars [13]. However, SNP marker technology requires special equipment and it is costly. In contrast, SSR markers are very convenient and easy to use. SSR markers are tandemly repeated nucleotide sequence motifs [14]; that are dispersed throughout the genome and may exhibit locus-specific codominance and high polymorphism rates, and have a high level of transferability [15,16,17]. Furthermore, molecular markers can be used to distinguish cultivars or plants originating from different locations. SSR markers are effective tools that play an important role in plant cultivar identification and genetic diversity analysis [18]. The next generation sequencing (NGS) technologies can be used for fast and cost-effective SSR discovery in many crops [19].

The *Angelica* species used in conventional medicine varies by country according to specific regulations, i.e., *A. gigas* in Korea, *A. sinensis* in China, and *A. acutiloba* in Japan. Because of the similarity between the names among *Angelica*, they can be confused in the market [20]. SSR markers are especially useful to distinguish closely related genotypes. This is the reason that SSR markers are widely used in genetic studies and identification of closely related cultivars. By using these SSR markers, various problems of *Angelica* genus can be solved [21].

In this study, we developed polymorphic SSR markers from several *A. gigas* accessions using NGS and bioinformatics approaches. The SSR markers developed in this study could be used for genetics and the breeding of *Angelica* genus.

## 2. Material and Methods 

### 2.1. Plant Material and DNA Isolation 

Sixteen widely cultivated *A. gigas* accessions were collected in Korea (Table S1). All of the collected roots or seeds were germinated and grown in the Chungbuk National University greenhouse. Young leaves or fresh seedlings were used for extraction of the genomic DNA (gDNA), which is required for library construction and sequencing. Fresh leaves were ground with liquid nitrogen and DNA was extracted using a DNeasy Plant Mini kit (Qiagen, Valencia, CA, USA) according to the manufacturer’s instructions.

### 2.2. Genomic DNA Sequencing and Sequence Assembly

The libraries were constructed from five *A. gigas* accessions: Manchu variety (MV), Hwangje variety (HV), Gangwon local variety (GLV), Jecheon local variety (JLV), and Sancheong local variety (SLV). Sequencing size was one lane for MV and 1/3 lane for the other four varieties. Sequencing libraries for DNA samples were prepared by a TruSeq Nano DNA Sample Prep Kit, according to the manufacturer's instructions (Illumina, Inc., San Diego, CA, USA). The libraries were subjected to paired-end sequencing with a 101 bp read length using the Illumina HiSeq 2500 platform (Illumina). Errors of short reads for each sample were corrected by using the SOAPec part of SOAPdenovo2 version 2.04 [22]. After error correction, short reads corresponding to ≥13× of genome size, estimated on the basis of a *k*-mer frequency spectrum, were assembled by using SOAPdenovo2 with the adjustment of *k*-mer size of 53. Repetitive DNA sequences, such as microsatellites, transposable elements, and rDNAs, were screened in the assembled contigs by using RepeatMasker version 4.0.5 [23] and RepeatModeler version 1.0.8 [23]. From the resulting repeat-masked sequences, gene models were built by ab initio prediction coupled with transcript alignment using AUGUSTUS version 3.1 [24], with the training gene set derived from the genome of *Platycodon grandiflorum* (unpublished), which belongs to the subclass Asterids. Genes were searched against UniProt and NCBI non-redundant (NR) protein databases using BLASTX, with a cutoff *E*-value of 10^−10^. Protein domains in genes were searched by using InterProScan version 5.17-56.0 [25].

### 2.3. SSR Findings and Primer Designs 

Simple sequence repeats were identified by using SSR Finder [26] with the following parameters: (1) microsatellites consisting of tandem repeats of 2 to 10 bp with a minimum of five repeats; and, (2) no variation (mutation) in repeat motifs was permitted. Primers were designed from flanking sequences of SSR microsatellite loci by using Primer3 version 2.2.3 [27] with the following parameters: primer length = 18–26 bp (Opt. 23 bp); GC % = 50–70% (Opt. 60%); temperature = 55–62 °C (Opt. 58 °C); and, product size range = 150–200 bp. Repetitive DNA, as well as duplicate sequences, were excluded and selected primers from the unique genomic regions. Forward primers were labeled with a virtual dye 6-FAM, NED, VIC, or PET (Applied Biosystems, Foster City, CA, USA). 

### 2.4. In Silico SSR Polymorphism Screening 

In silico SSR polymorphism screening was performed by using CLC Main Workbench version 6.8.4 (CLC bio, Aarhus, Denmark) BLAST tool. HV, GLV, JLV and SLV sequences were assembled by using CLC Genomics Workbench version 7.5 (CLC bio, Aarhus, Denmark) and prepared for BLAST data base. The SSR regions (450 bp each) identified from MV sequences were used as reference sequences in a BLAST search. BLAST analyses were performed by the following parameters: BLAST program = blastn: DNA sequence and database; Target = BLAST database (HV, GLV, JLV and SLV sequences); Number of threads = 1; Choose filter = Filter low complexity; Expect = 10.0; Word size = 3; Match/mismatch cost = match 1, mismatch −3; Gap costs = Existence 5, Extension 2; and, Max number of hit sequences = 250 (Figure S1).

### 2.5. PCR Amplification and Genotyping

The PCR reaction mixture (20 μL) consists of 20 ng of gDNA, 1× HSTM Taq DNA polymerase buffer, 1.5 mM MgCl2, 0.2 mM of each dNTP, 0.2 M of each primer, and 1.25 units HSTM Taq DNA polymerase (Dongsheng, Göttingen, Germany). DNA amplification was performed by using the PCR system (BIO-RAD T-100 Thermal Cycler) according to the following: 5 min for initial denaturation at 95 °C, 34 cycles of 30 s at 94 °C, 30 s at 58–60.5 °C, 30 s at 72 °C, concluding with 1 cycle of 30 min at 72 °C. PCR products were separated in a 4% agarose gel to check the amplification and 0.2 L of PCR products were mixed with 9.8 L Hi-Di formamide (Applied Biosystems, Foster City, CA, USA), and 0.2 L of GeneScanTM 500 LIZ^®^size standard (Applied Biosystems). The PCR product mixture was denatured at 95 °C for 5 min and kept on ice. Subsequently, the mixture was separated by capillary electrophoresis on the ABI 3730 DNA analyzer (Applied Biosystems) by using a 50 cm-capillary array with a DS-33 install standard as a matrix. We analyzed the amplified fragments by size with GeneMapper software version 4.0 (Applied Biosystems). The number of alleles (*N_A_*), frequency of major alleles (*M_AF_*), observed heterozygosity (*H_O_*) values, expected heterozygosity (*H_E_*) values, and polymorphic index content (PIC) values were measured by using PowerMarker software version 3.23 [28]. Coefficients of genetic similarity were calculated by using the molecular evolutionary genetics analysis (MEGA) program version 5.05 [29]. Genetic distance was computed by using the SharedAllele distance method with PowerMarker software version 3.23. 

## 3. Results and Discussion

### 3.1. Characteristics of Genomic SSR in the NGS Assemblies of A. gigas

Libraries were constructed from the five *A. gigas* accessions (Table 1) and subjected to paired-end sequencing with a 101 bp read length by using an Illumina HiSeq 2500 platform. The SOAPdenovo2 assembler (version 2.04) was used to assemble the *A. gigas* genome de novo, generating 395,007 scaffolds representing 804 Mb from the *A. gigas* (MV) genome. MV is a breed that was developed based on the root hypertrophy and slow-bolting characteristic from *A. gigas*. Thus, we selected MV as a representative *A. gigas* variety. The other four varieties were selected due to their diverse geographical origins. The four varieties were used for in silico polymorphism analysis. Analysis of *k*-mer frequency spectrum revealed a peak (*k*-mer depth: 11) in the 17 *k*-mer depth distribution of reads, suggesting a low heterozygosity across the genome (Figure S2). Based on the *k*-mer frequency analysis, the genome size of MV was estimated to be approximately 2.67 Gb (No. of *k*-mer/depth of *k*-mer at peak). Conclusively, we found, 138,113 SSRs from the de novo assembly of the MV genome. The total number of 121,112 di-nucleotide, and 13,211 tri-nucleotide SSRs constituted 87.691% and 9.565% of SSRs, respectively, followed by tri-, tetra-, penta- and hexa-nucleotide motifs (Table S2).

### 3.2. SSR Marker Development by In Silico Polymorphism Analysis and Characterization of SSR Markers 

We successfully designed 16,496 SSR primer pairs, out of 138,113 SSRs from the unique genomic regions of MV genome assembly. The majority of designed SSR primer sets was di-nucleotide (14,113, in SSR, 10.218%), followed by tri-nucleotide (2064, in SSR, 1.494%), tetra-nucleotide (242, in SSR, 0.175%), penta-nucleotide (36, in SSR, 0.026%), and hexa-nucleotide (37, in SSR, 0.027%) motifs (Table S2). Initially, five *A. gigas* accessions (MV, HV, GLV, JLV, and SLV) were selected to detect the polymorphism of these primer sets by in silico analysis. 

The MV scaffolds including the SSRs were subjected to BLAST analysis against the assembled contigs of four other varieties (HV, GLV, JLV, and SLV). Polymorphic SSR markers showed two to five different repeat length scaffolds from the five *A. gigas* accessions. Consequently, a total of 848 polymorphic SSR markers were selected from the in silico analyzed tri-nucleotide to hexa-nucleotide motifs (a total of 2379 SSR primer sets).

We selected 48 polymorphic SSR markers, from 848 in silico analyzed primer pairs, which were showing four or more different repeat length scaffolds types, or more than 15 bp of nucleotide length difference (Table S2; Figure S1) and further used for genotyping of five *A. gigas* accessions (MV, HV, GLV, JLV, and SLV). Thirty-six SSR primer sets yielded balanced, intact, reproducible, and polymorphic amplicons in 4% agarose gels. The remaining 12 SSR primer sets were amplified more than two bands or showed unexpected amplicon sizes. The 36 SSR primer sets were deposited to GenBank (Table 2). Genomic regions of the 36 SSR loci were also analyzed on the basis of ab initio gene identification in the assembled sequences, and 16 (44.4%), 9 (25.0%), 9 (25.0%), and 2 (5.6%) were found from intergenic, intron, coding DNA sequence (CDS), and 5′ untranslated region (5'UTR), respectively (Table S3). 

Subsequently, 16 *A. gigas* accessions were used to detect the polymorphisms of the 36 SSR loci after one of the primers from each primer set was labeled. Polymorphism estimation indicated that the *N_A_* per locus varied widely among the markers, ranging from 3 to 15, with an average of 6.36 alleles. The *M_AF_* per locus was 0.16–0.63, with an average of 0.39. In addition, the *H_E_* values were 0.53–0.90, with an average of 0.73; *H_O_* values were 0.13–0.88, with an average of 0.53. Ultimately, PIC values were 0.44–0.89, with an average of 0.69 (Table 3). Thirty-six SSR markers showed a high PIC value (PIC > 0.6) and, based on our results, we constructed the genetic relationship of 16 *A. gigas* accessions. Phylogenetic trees were constructed for the 16 *A. gigas* accessions by unweighted pair group method with arithmetic mean (UPGMA) analysis (Figure S3). 

The study of genetic diversity and genetic relationships of crops can assist breeders in the development of economically effective cultivars. SSR markers have been used in many areas, including linkage map development, QTL mapping, marker-assisted selection, cultivar fingerprinting, genetic diversity, gene flow, and evolutionary studies in the botanic sciences [30,31,32]. NGS technology is a modern technique used to discover large-scale genetic polymorphisms [32,33,34]. Since NGS technology allows cost-effective and higher throughput genome sequencing, it has been universally applied for many crop species, including *Allium fistulosum* L., *Sesamum indicum* L., and *Vicia sativa* subsp. *Sativa* [35,36]. Recently, a study on the development of 18 polymorphic microsatellite markers for *A. sinensis* using NGS technology has been reported although it was focused on species identification [37]. In this study, we developed SSR markers that could be used for genetics, breeding, and diversity analyses of *Angelica* species. 

The selection of isolated SSR loci from the genome or transcriptome data would be useful to analyze data, project goals, and future plans [34]. Our mass production method of SSR markers using an in silico approach, and resulted in 36 new polymorphic SSR markers from *A. gigas*. The developed markers from this study are expected to be useful in crop genetics and breeding. Even though the remaining 12 SSR markers amplified multiple bands or yielded bands of unexpected size, they were also polymorphic in the sequence. Therefore, the 848 SSR marker candidates discovered through this research are expected to represent informative markers. 

Even though we restricted the purpose of this study on the selection of polymorphic SSR markers for the genetic diversity analysis of the genetic resources, application of the primers to more diverse *Angelica* species resources can enlarge the usage of the developed markers. In addition, we hope that the *A. gigas* sequence data of this study can be used to develop various molecular markers, such as SNP and insertion-deletion (InDel) markers, in the future.

## Figures and Tables

**Table 1:** Genome sequencing of five *Angelica gigas* accessions.

	MV	HV	GLV	JLV	SLV
Total Reads	351,885,410	153,890,676	112,354,576	139,011,848	142,791,294
Total Bases	35,540,426,410	15,542,958,276	11,347,812,176	14,040,196,648	14,421,920,694
Number of Scaffolds	395,007	541,229	468,810	526,114	546,442
Assembled genome size (bp)	804,583,850	298,362,936	242,460,776	273,337,815	298,605,080

MV, Manchu variety; HV, Hwangje variety; GLV, Gangwon local variety; JLV, Jecheon local variety; SLV, Sancheong local variety.

**Table 2:** Characterization of 36 polymorphic single sequence repeat (SSR) markers validated in 16 *A. gigas* accessions.

Marker	GenBank No.	Motif	Primer Sequence	Annealing Temperature (°C)	Allele Size (bp)
YL-AGN tri0110	KX138563	(AAT) 5	F:FAM-TATGTCGGCAACACATGC	58	177–186
			R:GTTTGGCTAGGCTAGACTAATGTGGGT		
YL-AGN tri0221	KX138564	(ACC) 7	F:FAM-CGACACCAGTGTTGATCCAT	58	179–194
			R:GTTTGTATCGAGTCCATAACGCTCAG		
YL-AGN tri0303	KX138565	(AGC) 5	F:FAM-AGGCGATAGAATCCCCAA	58	157–175
			R:GTTTCGAGAAACAAAGCTTCAGGG		
YL-AGN tri0336	KX138566	(AGG) 8	F:FAM-GTGGTGTTCTTTTCTCCACG	58	185–203
			R:GTTTCTTGTCGTCTTCTCGTCTCTCT		
YL-AGN tri0359	KX138567	(AGT) 8	F:FAM-CGTCGGCTAGTAACTGCAAA	58	188–194
			R:GTTTATGTCGCGGTGATTTACG		
YL-AGN tri0379	KX138568	(ATA) 15	F:FAM-GCTGTTCTGGCGTATGTACTTC	58	161–191
			R:GTTTACTCCCAACAAACACTGCTC		
YL-AGN tri0537	KX138569	(ATG) 7	F:FAM-GGTCCTCAGCTTTCTCAGAATC	58	175–217
			R:GTTTCCTGATATCTCCCTGCAATC		
YL-AGN tri0638	KX138594	(ATT) 7	F:PET-AGCATAGGATCCAGCTTGTG	58	134–161
			R:GTTTTTTCGAGATGGAGTCTCAGC		
YL-AGN tri0685	KX138570	(CAC) 5	F:FAM-AGTAAGCAAGTGATGCCGAG	58	179–197
			R:GTTTGGGGGTTTTGTAGTGGTTCA		
YL-AGN tri0832	KX138571	(CCA) 5	F:FAM-CCACTTTACTCCCCTGATAAGC	58	163–184
			R:GTTTTTCCTGCCAGCTCTGATTAC		
YL-AGN tri0861	KX138572	(CCT) 5	F:NED-CTGGTGGCTCTTAAGTTACTCG	58	193–230
			R:GTTTCGGTTACTACAGTACGTTGGTTGC		
YL-AGN tri0889	KX138573	(CCT) 5	F:NED-GACAAAGAAGCAGTGGCATC	58	193–202
			R:GTTTGAGATTACGACGAGCGAGAA		
YL-AGN tri0953	KX138595	(CTG) 16	F:PET-TGTTTGCACCAGCTCTCA	58	180–209
			R:GTTTCCACCTCAAGGTTCAATGAC		
YL-AGN tri0955	KX138596	(CTG) 9	F:PET-TAGCCAAGACCAGCTCAATC	58	144–167
			R:GTTTTACTGCTACAATGGCACACC		
YL-AGN tri0957	KX138574	(CTG) 6	F:NED-CAGTTCCGTTGTTCCAACTC	58	188–203
			R:GTTTGAAAGCGAACGAGAGATGAG		
YL-AGN tri1043	KX138575	(GAA) 8	F:NED-GCTGGATTATCACCTTCACG	58	191–204
			R:GTTTGAAGAGGTAATGTGGGGTGTAG		
YL-AGN tri1092	KX138597	(GAA) 14	F:PET-CTAGCTTTCCCATGTCTGAACC	58	143–201
			R:GTTTCATCCATGCACCAATGTC		
YL-AGN tri1102	KX138576	(GAC) 5	F:NED-ACTCAAAAGGACAAGTCCCC	58	150–200
			R:GTTTCCTTCCACTCTCTGGTTGTAGAC		
YL-AGN tri1118	KX138577	(GAG) 5	F:NED-AACTGATTGGGAGGAGTAGGAG	58	168–183
			R:GTTTCCTGAAAGAGTACTAACACCCG		
YL-AGN tri1174	KX138578	(GAT) 6	F:NED-AGCAACTAGCTCACTCACAACC	58	196–210
			R:GTTTAGACGAGTTAAGGGACTTGC		
YL-AGN tri1211	KX138579	(GCA) 8	F:NED-ATGCAACAATGTCTCGGC	58	162–180
			R:GTTTCAACTTGGGTTCTGCCCTAT		
YL-AGN tri1262	KX138580	(GCT) 7	F:NED-GTTACATTGAGTCCTCGTAGGG	58	171–199
			R:GTTTATCACGAACCAGAACCCA		
YL-AGN tri1269	KX138581	(GCT) 5	F:VIC-CCCTTGACACTCACTACTCTCA	58	202–217
			R:GTTTTTCTCTCTGCTACCCTTAGAGC		
YL-AGN tri1276	KX138598	(GCT) 5	F:PET-GATTGCTGCTGCTGAGTTG	58	156–177
			R:GTTTGAACTCTCCGATGGTGTTGTAG		
YL-AGN tri1358	KX138582	(GTA) 6	F:VIC-GACAGCCAGCCTTCTTCAT	58	182–194
			R:GTTTCCCTACATTGCGTTGATCC		
YL-AGN tri1582	KX138583	(TCA) 8	F:VIC-GGTACGGGAATATGAGACAGG	58	183–206
			R:GTTTGTGATCTTGCTGATGACGG		
YL-AGN tri1708	KX138584	(TCT) 10	F:VIC-GTGGTGCTGCCAATGTTT	58	172–241
			R:GTTTGAAGATGTCGGCAGATCAGT		
YL-AGN tri1849	KX138585	(TGG) 7	F:VIC-GTCCAATGATTCTGCTGCTC	60.5	174–183
			R:GTTTATATACTGGGAGTGTTGCGGAG		
YL-AGN tri1920	KX138586	(TTA) 8	F:VIC-TACAGTCGAGTTCTGGACACAC	58	179–200
			R:GTTTCTTTCTCCGTGAACATGTCG		
YL-AGN tetra2116	KX138587	(AGAT) 9	F:VIC-GTCACTAAAACACGAGACTGCC	58	179–250
			R:GTTTCCAGAACTCGTTCCGAATC		
YL-AGN tetra2173	KX138588	(ATTT) 5	F:VIC-AGGCATGCACTACTCCTCTATC	58	179–191
			R:GTTTATCTCGCAGCTATGAGACTACC		
YL-AGN tetra2208	KX138589	(GATA) 6	F:VIC-GCTGGGTTTAGGTTTCTGGA	60.5	157–177
			R:GTTTACCGCAAACACCTAGTACTCC		
YL-AGN tetra2275	KX138590	(TGTA) 7	F:PET-CATCATCTTGCAAGGTCCAC	58	164–188
			R:GTTTTCTCCCAAACTGGTACTCTG		
YL-AGN Hexa2350	KX138591	(AGAATC) 6	F:PET-GCTAGCAATAGCAGGTTGAC	58	193–210
			R:GTTTATAGACCTGGTTTCGGGC		
YL-AGN Hexa2367	KX138592	(GATCTC) 5	F:PET-ACGTGACCACCATATTGC	58	187–199
			R:GTTTGTGGACACTGTTTCGTCACTG		
YL-AGN Hexa2374	KX138593	(TCATGC) 5	F:PET-GGCAGACGTTCTGGTTTTC	58	164–176
			R:GTTTCCGTGAGTGGTAGGGAAATA		

**Table 3:** Diversity statistics from initial primer screening in 16 *A. gigas* accessions.

No.	Marker	*M_AF_*	*N_G_*	*N_A_*	*H_E_*	*H_O_*	PIC
1	YL-AGNtri0110	0.47	7	4	0.68	0.67	0.63
2	YL-AGNtri0221	0.25	11	6	0.80	0.63	0.77
3	YL-AGNtri0303	0.50	8	5	0.67	0.50	0.62
4	YL-AGNtri0336	0.34	11	5	0.77	0.50	0.73
5	YL-AGNtri0359	0.57	4	3	0.56	0.33	0.48
6	YL-AGNtri0379	0.28	10	7	0.78	0.44	0.75
7	YL-AGNtri0537	0.53	9	7	0.67	0.56	0.64
8	YL-AGNtri0638	0.33	9	5	0.75	0.40	0.71
9	YL-AGNtri0685	0.44	10	7	0.73	0.69	0.70
10	YL-AGNtri0832	0.38	8	7	0.76	0.19	0.73
11	YL-AGNtri0861	0.31	9	10	0.84	0.50	0.82
12	YL-AGNtri0889	0.59	4	3	0.53	0.19	0.44
13	YL-AGNtri0953	0.31	13	10	0.82	0.75	0.81
14	YL-AGNtri0955	0.44	8	5	0.72	0.69	0.68
15	YL-AGNtri0957	0.31	9	5	0.75	0.44	0.71
16	YL-AGNtri1043	0.63	7	5	0.56	0.44	0.52
17	YL-AGNtri1092	0.22	15	11	0.87	0.63	0.86
18	YL-AGNtri1102	0.34	11	8	0.79	0.44	0.77
19	YL-AGNtri1118	0.34	9	4	0.71	0.69	0.66
20	YL-AGNtri1174	0.38	9	5	0.71	0.31	0.66
21	YL-AGNtri1211	0.41	10	6	0.75	0.50	0.71
22	YL-AGNtri1262	0.31	10	8	0.80	0.88	0.77
23	YL-AGNtri1269	0.50	8	6	0.68	0.75	0.64
24	YL-AGNtri1276	0.25	13	8	0.84	0.56	0.82
25	YL-AGNtri1358	0.47	9	5	0.67	0.44	0.62
26	YL-AGNtri1582	0.31	11	8	0.82	0.75	0.80
27	YL-AGNtri1708	0.16	14	14	0.90	0.50	0.89
28	YL-AGNtri1849	0.63	3	4	0.54	0.13	0.48
29	YL-AGNtri1920	0.31	13	8	0.81	0.75	0.79
30	YL-AGNtri2275	0.41	9	6	0.73	0.69	0.69
31	YL-AGNtetra2116	0.22	13	14	0.88	0.75	0.87
32	YL-AGNtetra2173	0.41	8	4	0.69	0.75	0.63
33	YL-AGNtetra2208	0.41	7	5	0.67	0.25	0.61
34	YL-AGNhexa2350	0.44	7	4	0.64	0.56	0.57
35	YL-AGNhexa2367	0.46	6	4	0.64	0.38	0.57
36	YL-AGNhexa2374	0.44	6	3	0.63	0.44	0.56
	Mean	0.39	9.1	6.4	0.73	0.53	0.69

*M_AF_*, major allele frequency; *N_G_*, number of genotypes; *N_A_*, number of alleles; *H_E_*, expected heterozygosity; *H_O_*, observed heterozygosity; PIC, polymorphism information content.

## Supplementary Materials

The following are available online at www.mdpi.com/2073-4425/8/10/238/s1. Figure S1: Different repeat length scaffold types of four or more, and SSR markers of more than 15 bp nucleotide length difference, Figure S2: The *k*-mer depth distribution of whole-genome sequencing reads of *Angelica gigas*, Figure S3: Dendrogram generated using UPGMA cluster analysis based on genetic diversity of 16 *Angelica gigas* Nakai accessions, Table S1: List of 16 *Angelica gigas* accessions and information of collection sites, Table S2: Details of Manchu variety accession repeat genome assemblies, Table S3: Number of designed SSR primer sets and polymorphic SSRs discovered in silico.
