# Supplementary material for: Development of Genome-Wide SSR Markers from Angelica gigas Nakai Using Next Generation Sequencing
Source: Genes (Basel). 2017 Sep 21;8(10):238. doi: 10.3390/genes8100238 (PMC5664088; doi:10.3390/genes8100238)
Supplement: Supplementary file 1 [file genes-08-00238-s001.docx]

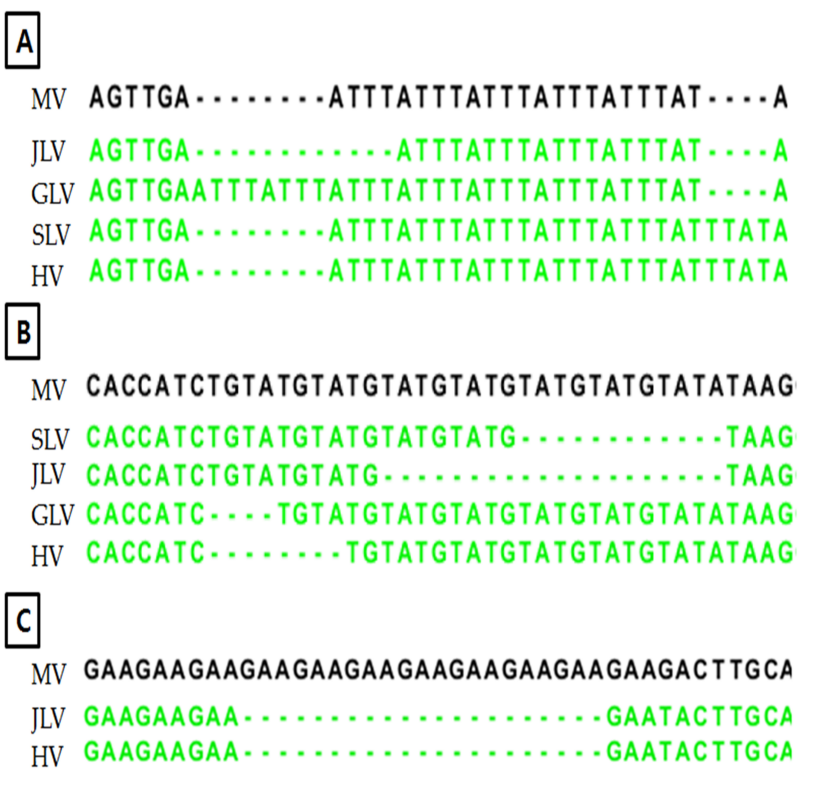
**Figure S1.** Different repeat length scaffold types of 4 or more and SSR markers of more than 15 bp nucleotide length difference. (A) Four different repeat length scaffold type of YL-AGN tetra2173 marker; (B) five different repeat length scaffold of YL-AGN tetra2116 marker; (C) twenty-one bp nucleotide length difference of YL-AGN tri1092 marker.


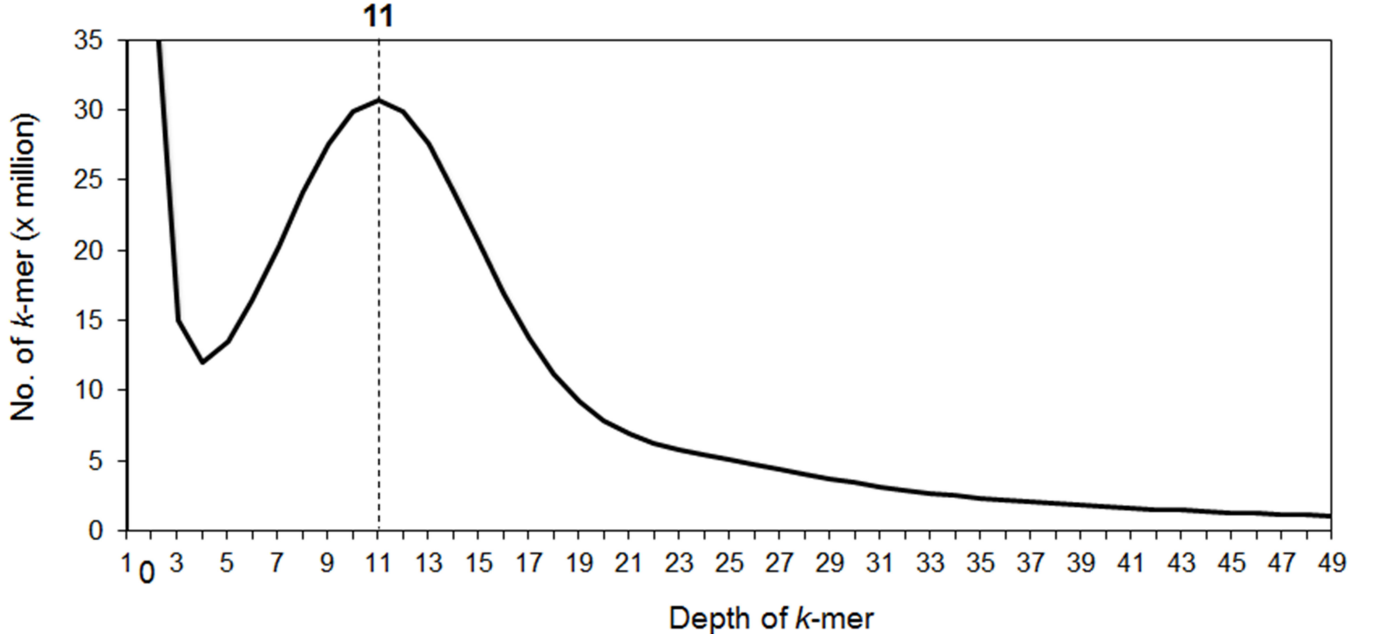


**Figure S2.** The *k*-mer depth distribution of whole-genome sequencing reads of *Angelica gigas*.


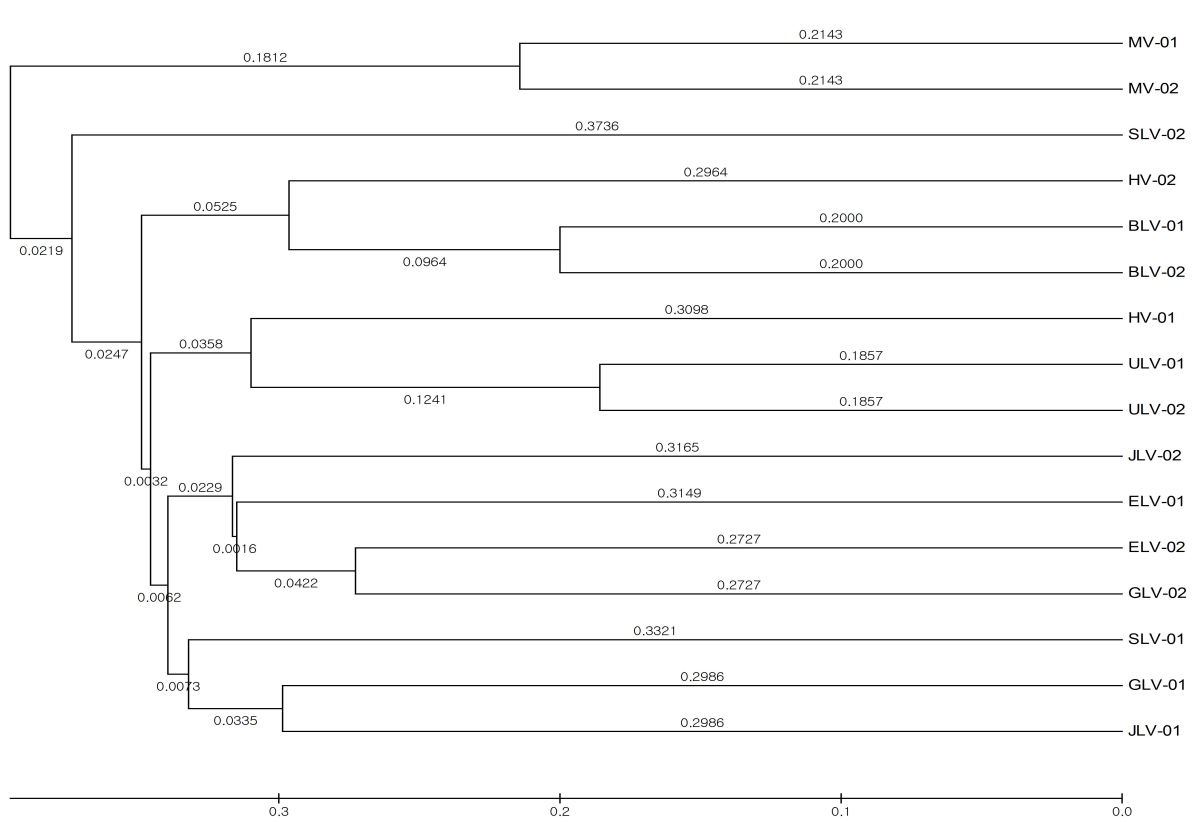


**Figure S3.** Dendrogram generated using UPGMA cluster analysis based on genetic diversity of 16 *Angelica gigas* Nakai accessions. MV, Manchu variety; SLV, Sancheong local variety; HV, Hwangje variety; BLV, Bonghwa local variety; ULV, Seongbuk local variety; JLV, Jecheon local variety; ELV, Eumseong local variety; GLV, Gangwon local variety.

**Table S1.** List of 16 *Angelica gigas* accessions and information of collection sites.

| **No.** | **Accessions Code Name** | **Collected region** |
| --- | --- | --- |
| 1 | HV-01 ***** | Jongno-gu, Seoul, South Korea |
| 2 | HV-02 |  |
| 3 | ULV-01 | Seongbuk-gu, Seoul, South Korea |
| 4 | ULV-02 |  |
| 5 | BLV-01 | Bonghwa-gun, Gyeongsangbuk-do(1), South Korea |
| 6 | BLV-02 |  |
| 7 | MV-01 ***** | Bonghwa-gun, Gyeongsangbuk-do(2), South Korea |
| 8 | MV-02 |  |
| 9 | JLV-01 ***** | Jecheon-si, Chungcheongbuk-do, South Korea |
| 10 | JLV-02 |  |
| 11 | SLV-01 ***** | Jiri Mountain, Gurye-gun, Jeollanam-do, South Korea |
| 12 | SLV-02 |  |
| 13 | GLV-01 ***** | Pyeongchang-gun, Gangwon-do, South Korea |
| 14 | GLV-02 |  |
| 15 | ELV-01 | Eumseong-gun, Chungcheongbuk-do, South Korea |
| 16 | ELV-02 |  |

HV, Hwangje variety; ULV, Seongbuk local variety; BLV, Bonghwa local variety; MV, Manchu variety; JLV, Jecheon local variety; SLV, Sancheong local variety; GLV, Gangwon local variety; ELV, Eumseong local variety; *, NGS sequencing accessions.

**Table S2.** Number of designed SSR primer sets and polymorphic SSRs discovered *in silico*.

| **Motif Type** | **No. of Searched SSRs** | **In SSR**  **(%)** | **No. of Primer designed SSRs** | **In SSR**  **(%)** | **No. of Polymorphic SSR markers** |
| --- | --- | --- | --- | --- | --- |
| Di | 121,112 | 87.691 | 14,113 | 10.218 | NT |
| Tri | 13,211 | 9.565 | 2,064 | 1.494 | 707 |
| Tetra | 3,039 | 2.200 | 242 | 0.175 | 113 |
| Penta | 433 | 0.314 | 36 | 0.026 | 12 |
| Hexa | 257 | 0.186 | 37 | 0.027 | 16 |
| Hepta | 46 | 0.033 | 3 | 0.002 | NT |
| Octa | 8 | 0.006 | 1 | 0.001 | NT |
| Ennea | 4 | 0.003 |  |  |  |
| Deca | 3 | 0.002 |  |  |  |
| Total | 138,113 |  | 16,496 | 11.944 | 848 |

NT, Not tested.

**Table S3.** Information about the putative function of the 36 polymorphic SSR marker loci of *Angelica gigas*.

| **Marker** | **Region** | **Description** |
| --- | --- | --- |
| YL-AGN tri0110 | Intergenic | - |
| YL-AGN tri0221 | Intron | Pumilio homolog 12, OS=*Arabidopsis thaliana*, GN=APUM12 |
| YL-AGN tri0303 | Intergenic | - |
| YL-AGN tri0336 | Intron | Ion channel CASTOR, OS=*Lotus japonicas*, GN=CASTOR |
| YL-AGN tri0359 | CDS | Unknown Gene |
| YL-AGN tri0379 | Intergenic | - |
| YL-AGN tri0537 | Intron | Bifunctional UDP-glucose 4-epimerase and UDP-xylose 4-epimerase 1, OS=*Pisum sativum*, GN=UGE1 |
| YL-AGN tri0638 | Intergenic | - |
| YL-AGN tri0685 | Intergenic | - |
| YL-AGN tri0832 | CDS | Early endosome antigen 1, OS=*Homo sapiens*, GN=EEA1 |
| YL-AGN tri0861 | Intron | ER lumen protein-retaining receptor, OS=*Entamoeba histolytica*, GN=ERD2 |
| YL-AGN tri0889 | Intergenic | - |
| YL-AGN tri0953 | Intron | GRF1-interacting factor 3, OS=*Arabidopsis thaliana*, GN=GIF3 |
| YL-AGN tri0955 | CDS | Transcription factor MYB98, OS=*Arabidopsis thaliana*, GN=MYB98 |
| YL-AGN tri0957 | CDS | Probable BOI-related E3 ubiquitin-protein ligase 3, OS=*Arabidopsis thaliana*, GN=BRG3 |
| YL-AGN tri1043 | Intergenic | - |
| YL-AGN tri1092 | Intergenic | - |
| YL-AGN tri1102 | CDS | Unknown Gene |
| YL-AGN tri1118 | CDS | Ethylene-responsive transcription factor ERF114, OS=*Arabidopsis thaliana*, GN=ERF114 |
| YL-AGN tri1174 | Intergenic | - |
| YL-AGN tri1211 | Intergenic | - |
| YL-AGN tri1262 | Intergenic | - |
| YL-AGN tri1269 | CDS | Ethylene-responsive transcription factor ERF003, OS=*Arabidopsis thaliana*, GN=ERF003 |
| YL-AGN tri1276 | CDS | TGACG-sequence-specific DNA-binding protein TGA-2.1, OS=*Nicotiana tabacum*, GN=TGA21 |
| YL-AGN tri1358 | CDS | Probably inactive leucine-rich repeat receptor-like protein kinase IMK2, OS=*Arabidopsis thaliana*, GN=IMK2 |
| YL-AGN tri1582 | Intergenic | - |
| YL-AGN tri1708 | Intergenic | - |
| YL-AGN tri1849 | 5'UTR | Alpha,alpha-trehalose-phosphate synthase [UDP-forming] 1, OS=*Arabidopsis thaliana*, GN=TPS1 |
| YL-AGN tri1920 | Intergenic | - |
| YL-AGN tetra2116 | Intron | Unknown Gene |
| YL-AGN tetra2173 | Intron | Protein ECERIFERUM 3, OS=*Arabidopsis thaliana*, GN=CER3 |
| YL-AGN tetra2208 | 5'UTR | Unknown Gene |
| YL-AGN tetra2275 | Intron | Thioredoxin H2, OS=*Arabidopsis thaliana*, GN=TRX2 |
| YL-AGN Hexa2350 | Intergenic | - |
| YL-AGN Hexa2367 | Intergenic | - |
| YL-AGN Hexa2374 | Intron | Mitogen-activated protein kinase kinase 2, OS=*Arabidopsis thaliana*, GN=MKK2 |

CDS, coding DNA sequence; GN, gene name; OS, organism name; UTR, untranslated region.
